# CAMR: cross-aligned multimodal representation learning for cancer survival prediction

**DOI:** 10.1093/bioinformatics/btad025

**Published:** 2023-01-13

**Authors:** Xingqi Wu, Yi Shi, Minghui Wang, Ao Li

**Affiliations:** School of Information Science and Technology, University of Science and Technology of China, Hefei AH230027, China; School of Information Science and Technology, University of Science and Technology of China, Hefei AH230027, China; School of Information Science and Technology, University of Science and Technology of China, Hefei AH230027, China; School of Information Science and Technology, University of Science and Technology of China, Hefei AH230027, China

## Abstract

**Motivation:**

Accurately predicting cancer survival is crucial for helping clinicians to plan appropriate treatments, which largely improves the life quality of cancer patients and spares the related medical costs. Recent advances in survival prediction methods suggest that integrating complementary information from different modalities, e.g. histopathological images and genomic data, plays a key role in enhancing predictive performance. Despite promising results obtained by existing multimodal methods, the disparate and heterogeneous characteristics of multimodal data cause the so-called modality gap problem, which brings in dramatically diverse modality representations in feature space. Consequently, detrimental modality gaps make it difficult for comprehensive integration of multimodal information via representation learning and therefore pose a great challenge to further improvements of cancer survival prediction.

**Results:**

To solve the above problems, we propose a novel method called cross-aligned multimodal representation learning (CAMR), which generates both modality-invariant and -specific representations for more accurate cancer survival prediction. Specifically, a cross-modality representation alignment learning network is introduced to reduce modality gaps by effectively learning modality-invariant representations in a common subspace, which is achieved by aligning the distributions of different modality representations through adversarial training. Besides, we adopt a cross-modality fusion module to fuse modality-invariant representations into a unified cross-modality representation for each patient. Meanwhile, CAMR learns modality-specific representations which complement modality-invariant representations and therefore provides a holistic view of the multimodal data for cancer survival prediction. Comprehensive experiment results demonstrate that CAMR can successfully narrow modality gaps and consistently yields better performance than other survival prediction methods using multimodal data.

**Availability and implementation:**

CAMR is freely available at https://github.com/wxq-ustc/CAMR.

**Supplementary information:**

Supplementary data are available at *Bioinformatics* online.

## 1 Introduction

Cancer has been considered as one of the major public health issues worldwide. It is reported that the cancer incidence will be increasing in the next decades and 420 million new cases of cancer are expected annually by 2025 (Wang *et al.*, 2021a). Due to the complexity of cancer, there are striking differences in not only molecular patterns but also clinical characteristics among cancer patients (Beck, 2015), leading to a major hurdle for appropriate therapy and accurate prognosis. Therefore, accurate cancer survival prediction plays a key role in helping clinicians to plan treatments (Gao *et al.*, 2021; Sun *et al.*, 2019).

In the medical field, survival prediction mainly focuses on modeling the elapsed time from the beginning of follow-up to the occurrence of an event of interest (Chi *et al.*, 2021). During the past years, many approaches have been proposed to address the problem of predicting cancer survival. Among these approaches, one of the research hotspots is to utilize histopathological images for survival prediction (Shao *et al.*, 2020). Histopathological images can reflect the underlying molecular processes and disease progression of cancer and thus are regarded as the gold standard for cancer prognosis (Shao *et al.*, 2021). To fully exploit the hidden information of histopathological images, a number of computational approaches (Di *et al.*, 2022; Yao *et al.*, 2020; Zhu *et al.*, 2017) have been introduced to retrieve a large amount of features from histopathologic images, which quantitatively reflect the size, shape, distribution and texture of cells. Alternatively, some other approaches (Huang *et al.*, 2019; Lee and Lim, 2019; Sun *et al.*, 2019) take advantage of genomic data to predict cancer survival, since cancer is strongly associated with genomic mutations or abnormal gene expression which alter normal cellular functions and biological processes (Shao *et al.*, 2020). Consequently, exploring the genetic information is of great significance for cancer survival prediction. For example, as a key component of genetic variation, copy number alteration (CNA) shows great potential for cancer survival prediction (Gao *et al.*, 2021; Sun *et al.*, 2019). Besides, many studies have successfully identified prognostic factors in cancer from gene expression data and achieved good performance for survival prediction (Huang *et al.*, 2019; van Wieringen *et al.*, 2009).

Given the importance of the aforementioned cancer-related data, systematic investigation of histopathological images and genomic data from a multimodal perspective, can provide a better understanding of the mechanism for cancer development and powerful computational tools for cancer survival prediction. Over the past years, machine learning methods (Cheng *et al.*, 2017; Yuan *et al.*, 2012; Zhang *et al.*, 2019; Zhao *et al.*, 2022) using multimodal data have been widely applied to make survival prediction. For example, Yuan *et al.* (2012) use SVM with a Gaussian radial basis kernel to combine histopathological images and genomic data for better cancer prognosis prediction. Cheng *et al.* (2017) develop a Lasso-Cox model to improve the predictive performance by integrating both gene expression data and histopathological images. Zhang *et al.* (2019) propose a multiple-kernel learning method to predict the survival outcomes of lung cancer patients by making use of multimodal information of histopathological images and multi-omics data. Zhao *et al.* (2022) introduce an adaptive risk-aware sharable and individual subspace learning method to achieve promising performance of cancer survival prediction. Subramanian *et al*. (2021) utilize CCA to make full use of the correlations between histopathological images and genomic data. Dong *et al.* (2019) propose an efficient improved gcForest model named MLW-gcForest to solve the problem of adenocarcinoma staging by taking genetic data as inputs. Ning *et al.* (2021a) present a multi-constraint latent representation learning method called McLR to achieve promising performance of cancer prognosis by learning a common subspace. Besides, Ning *et al.* (2021b) introduce an impressive method called Relation-aware Shared Representation learning (RaSR) to unify both representation learning and prognosis modeling into a joint framework and improve the performance of cancer prognosis. The above studies confirm that the combination of multimodal data is very helpful to enhance the performance of survival prediction thus providing a solid foundation for further research.

Recently, several deep learning-based methods (Chen *et al.*, 2022; Mobadersany *et al.*, 2018; Wang *et al.*, 2021b; Zhan *et al.*, 2021) integrating data from different modalities have been proposed to further improve the performance of survival prediction. For example, Chen *et al*. (2022) present an integrated framework named Pathomic Fusion which fuses histopathology and genomic features by taking advantage of the Kronecker product to predict survival outcome. Our previous work (Wang *et al.*, 2021b) introduces a fusion method named GPDBN to enhance the predictive performance of cancer survival by considering both relations within and across different modalities. Zhan *et al.* (2021) propose an effective neural network model named two-stage Cox-nnet to make survival prediction by using histopathology images and gene expression data. Ning *et al.* (2020) develop a favorable cross-modal feature-based integrative framework, which combines deep-learning features extracted from images and eigengenes obtained by genomic data. Despite the promising results obtained by these studies, the disparate and heterogeneous multimodal data still remain a great challenge to survival prediction. In fact, heterogeneity in distinct data modalities may often bring in dramatically diverse representations in feature space, i.e. the so-called modality gap problem (Xu *et al.*, 2020). As a consequence, the existence of modality gaps will hamper the comprehensive integration of multimodal information, which greatly limits further improvements in the performance of cancer survival prediction.

Currently, many works have tried to weaken the heterogeneity in distinct data modalities, which can be summarized into three categories: coordinated representations, translation and alignment (Baltrusaitis *et al.*, 2019). For the coordinated representations, diverse representations are generated for each modality and constructed by coordination. Some works (Pan *et al.*, 2016; Xu *et al.*, 2015) utilize similarity models to minimize the distance between different modalities in the coordinated space. Although coordinated representations have obtained good performance, the coordinated space usually works for two modalities. Besides, translation which maps representation distributions from one modality to another plays a key role in reducing modality gaps and mining complementary information from different modalities. For example, Mai *et al.* (2020) propose an effective framework to match transformed distributions of all modalities and enhance the performance of sentiment analysis. Moreover, an alignment which is used to find relationships and correspondences between sub-components of instances from two or more modalities has been widely used to handle the issue of the heterogeneity of different representations. For example, a representation alignment framework named private-shared subspaces separation (P3S) is adopted by Xu *et al.* (2020) to generate cross-modal common representations and achieves remarkable performance.

Differing from other cancer survival prediction works, we mainly focus on the issue of modality gaps in this article, which is rarely mentioned in the current works of survival analysis. To address this problem, we propose a method called cross-aligned multimodal representation learning (CAMR) to generate both modality-invariant and -specific representations, which provide a comprehensive and disentangled view of the multimodal data. Effectively learning modality-invariant representations plays a key role in narrowing modality gaps, thus improving the performance of cancer survival prediction. In detail, CAMR introduces a cross-modality representation alignment learning network which aims towards reducing modality gaps by obtaining modality-invariant representations. By using adversarial training to transform distributions of one modality to those of another modality, CAMR projects the representations of all modalities into a common subspace to achieve cross-modality representation distributional alignment. Importantly, such representation alignment can efficiently learn modality-invariant representations and therefore bridge gaps among different modalities. Also, an efficient cross-modality fusion module named CMFM is proposed to further fuse modality-invariant representations into a unified cross-modality representation for each cancer patient. In addition to modality-invariant representations, CAMR also learns modality-specific representations which complement modality-invariant representations and therefore provide a holistic view of the multimodal data for cancer survival prediction. To validate the effectiveness of the proposed method, we evaluate CAMR on different datasets from the Cancer Genome Atlas (TCGA). Comprehensive experimental results demonstrate the power of CAMR for reducing modality gaps and improving the performance of cancer survival prediction.

To sum up, the main contributions of this work are 3-fold.

1. A novel comprehensive multimodal representation learning method called CAMR is proposed for cancer survival prediction. In order to reduce modality gaps, CAMR employs a cross-modality representation alignment learning network to effectively learn modality-invariant representations. Moreover, we propose a CMFM to fuse modality-invariant representations by modeling complex relations across different modalities.
2. A modality-specific representation learning network that contains three unique encoders is adopted to learn their modality-specific representations, which complement the modality-invariant representation. Taken together, these representations give a comprehensive view of the multimodal data, thus helping to further understand the complexity of cancer.
3. The experimental results on different datasets from TCGA demonstrate the power of CAMR for reducing modality gaps and achieving superior performance of cancer survival prediction.

## 2 Materials and methods

### 2.1 Data preprocessing

We test CAMR on three cancer datasets obtained from TCGA (Zhu *et al.*, 2014) including lower-grade glioma (LGG), breast invasive carcinoma (BRCA) and lung squamous cell carcinoma (LUSC), and more details are described in Supplementary Table S1. For each dataset, we select cancer patients whose digital whole-slide images, gene expression, CNA and the corresponding clinical data are all available. Besides, patient samples with extremely short (i.e. shorter than 30 days) or missing follow-up are excluded by following previous work (Cheng *et al.*, 2017). For comprehensive evaluation, we perform 5-fold cross-validation in experiments by following (Kim *et al.*, 2021; Shao *et al.*, 2021). To be specific, cancer patients are randomly divided into five subsets. For each round of training, four subsets are split into a training set (80%) and a validation set (20%), and the remaining subset is used for testing. We adopt training set to optimize the prediction model, and validation set is used to tune the hyperparameters. Then, we use C-index and AUC (Supplementary Methods) to assess the performance on the test set. The above experimental procedures are repeated for five rounds and the mean value and standard deviation of these metrics are reported.

In this study, the processing procedure of pathological images is as follows. First, by following previous work (Yu *et al.*, 2016), the whole-slide images captured at 40 magnifications are divided into tiles of 1000 *×* 1000 pixels by employing bftools under an open microscopy environment. Then, we calculate the summation of red, green and blue values of each tile to obtain image density, and 10 tiles with the highest image density are selected for further study (Sun *et al.*, 2018). Finally, we use CellProfiler (Carpenter *et al.*, 2006) to extract a total of 2343 image features, which contain the size, shape, texture as well as pixel intensity distributions of cells and nuclei. We perform *z*-score normalization and discretization to image features extracted from histopathological images.

Following previous study (Ding *et al.*, 2016), for genomic data, we remove the missing values appearing in more than 10% patients and utilize the weighted nearest neighbors algorithm to fill other missing values. We then process gene expression data to three categories: over-expression (1), baseline (0) and under-expression (−1) according to previous works (Gao *et al.*, 2021; Gevaert *et al.*, 2006). For CNA data, *z*-score is used to normalize the linear copy number values (Zhang *et al.*, 2018). Similar to Yu *et al.* (2019), the randomForestSRC package implemented by R is applied to select top 80 features from each modality, i.e. pathological image, gene expression and CNA for further study, respectively.

### 2.2 Cross-aligned multimodal representation learning

**Fig. 1.**
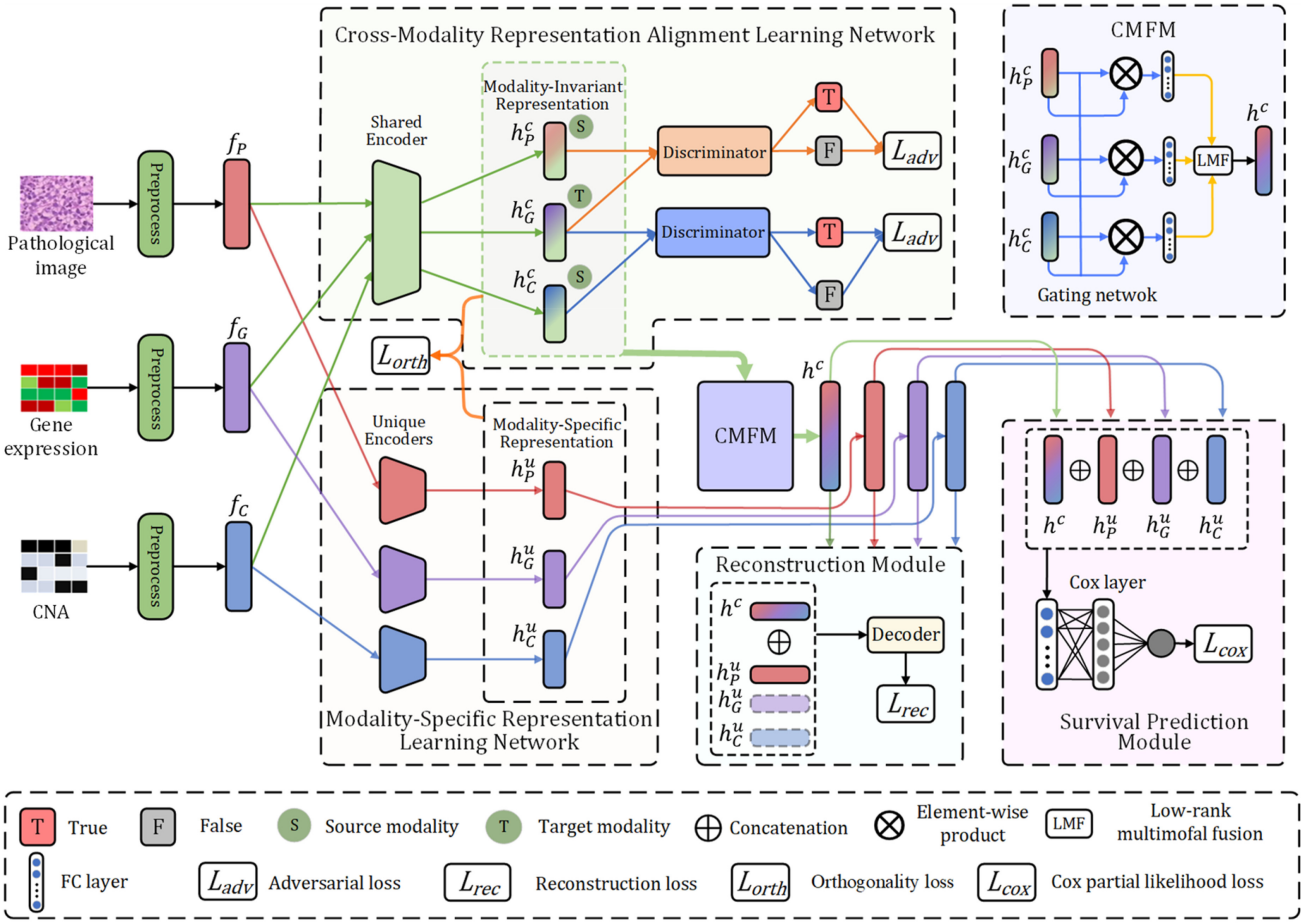
Illustration of the proposed CAMR framework

The overall framework of CAMR is shown in Figure 1. The cross-modality representation alignment learning network contains a shared encoder and two discriminators, which, by aligning the distributions of cross-modality representations through adversarial training, work together effectively to learn modality-invariant representations for reducing modality gaps. Following the construction of modality-invariant representations, CMFM is developed to fuse modality-invariant representations by modeling complex relations across different modalities. Besides, a modality-specific representation learning network includes three unique encoders dedicated to learn modality-specific representations, which provide complementary information to modality-invariant representations. Moreover, the fused modality-invariant representation and modality-specific representations are fed into a reconstruction module for better retaining modality information and learning comprehensive multimodal representations. Finally, by taking full advantage of the modality-invariant and -specific representations, a survival prediction module is employed to make accurate survival prediction.

#### 2.2.1 Cross-modality representation alignment learning

Since the diverse statistical properties among heterogeneous modalities make it difficult to provide comprehensive exploration of multimodal information, aligning distributions of different modality representations is crucial for reducing modality gaps and improving the performance of cancer survival prediction. With aim to eliminate modality gaps, in CAMR, we propose a cross-modality representation alignment learning network to map the representations of heterogeneous modalities into a common subspace, where discrepancies in modality representation distributions can be greatly alleviated in an adversarial way. Specifically, cross-modality representation alignment learning network establishes an adversarial game between the shared encoder and two discriminators (Mai *et al.*, 2020). The shared encoder performs the major task of learning modality-invariant representations in the common subspace and meanwhile tries to confuse the discriminators. Whereas the two discriminators seek to distinguish one modality from the other modalities, in this way steering the learning of modality-invariant representations.

Technically speaking, given the inputs of three modalities: histopathological image $f_{P}\in R^{p\times 1}$, gene expression $f_{G}\in R^{g\times 1}$ and CNA $f_{C}\in R^{c\times 1}$ (p denotes the dimensionality of $f_{P}$ and so on), CAMR tries to learn modality-invariant representations $h_{m}^{c},m\in \left\{P,G,C\right\}\;$via a shared encoder with a multi-layer fully connected (FC) architecture, which can be expressed as follows:

(1) hmc=Ecfm;θ,m∈P,G,C,

where$\;E_{c}\left(\cdot \right)$ denotes the encoder function shared across all modalities, θ represents parameters of $E_{c}$, and $\left\{P,G,C\right\}\;$represents three modalities histopathological image, gene expression and CNA. By using Equation (1), the representations of different modalities can be projected into a common feature subspace, and through optimizing the parameters θ, modality-invariant representations generated by the shared encoder are able to reduce the diversity across the representations of each modality and therefore narrow the modality gaps (Hazarika *et al.*, 2020).

To achieve this goal, we exploit adversarial training to help align the distributions of cross-modality representations in the common subspace. In detail, we choose gene expression as target modality due to its popularity in cancer survival prediction and the others as source modalities, with the aim to transform the representation distributions of the latter into those of the former. Then, we define two discriminators $D_{1}\;$and $D_{2}$, which act as adversaries and steer the shared encoder to learn better modality-invariant representations (Wang *et al.*, 2017). The first discriminator $D_{1}\;$tries to classify $h_{G}^{c}$ as true but $h_{P}^{c}$ as false. Similarly, the second discriminator $D_{2}$ assigns true label to $h_{G}^{c}$ and false label to $h_{C}^{c}$. Meanwhile, acting as a generator, the shared encoder seeks to fool $D_{1}\;$and $D_{2}$ to classify $h_{P}^{c}$ and $h_{C}^{c}$ as true. When two discriminators fail to distinguish target modality and source modalities, the distributions of different modality representations are efficiently aligned in the common subspace. Accordingly, the adversarial loss function${\;L}_{\mathrm{adv}}$ is composed of two parts: fake adversarial loss $L_{f}$ and true adversarial loss $L_{t}$, as shown below:

(2) Ladv=Lf+Lt,

where $L_{f}$ and $L_{t}$ are defined as follows:

(3)  Lf=-log⁡D1hPc-log⁡D2hCc,

(4) Lt=-log⁡1-D1hPc+log⁡D1hGc-log⁡1-D2hCc+log⁡D2hGc,

where $D_{1}\left(h_{P}^{c}\right)$ stands for the prediction value of $h_{P}^{c}\;$by $D_{1}$ and so on. By minimizing $L_{\mathrm{adv}}$ in Equation (2), the above adversarial training puts extra restrictions on the shared encoder and helps to better optimize the parameters θ. In this way, the proposed cross-modality representation alignment learning network can effectively learn modality-invariant representations and largely mitigate the problem of modality gaps.

#### 2.2.2 Cross-modality fusion module

By leveraging the cross-modality representation alignment learning network, modality-invariant representations can be obtained for histopathology images and genomic data. After that, CAMR employs CMFM to fuse them into a unified cross-modality representation by modeling hidden relations across modalities. Specifically, a gating network is first adopted as attention mechanism in CMFM to control the expressiveness of modality-invariant representations, which learns the weight vector $\alpha_{m}$ defined as follows:

(5) αm=wm*hPc⊕hGc⊕hCc,m∈P,G,C,

where $w_{m}$ is a learnable weight matrix and ⊕ refers to concatenation operator. We multiply modality-invariant representation with $\alpha_{m}$, and thus the output of the gating network is calculated as:

(6) h^mc=ReLUαm*h'mc,m∈P,G,C,

where ${h'}_{m}^{c}$ is the output of a FC layer using$\;h_{m}^{c}$ as input, ReLU represents active function. Then, to generate the cross-modality representation $h^{c}\in R^{d\times 1}$, we utilize low-rank multimodal fusion (LMF) (Liu *et al.*, 2018) to fully exploit intrinsic relations across modality-invariant representations, which can be formulated as:

(7) hc=⋀m∑i=1rwmi·h^mc,m∈P,G,C,

where Λ denotes the element-wise product, $w_{m}^{\left(i\right)}$ is a learnable low-rank decomposition matrix, and *r* is the total number of decomposition matrices. The basic idea of Equation (7) is to seek a set of low-rank decomposition matrices to fuse tensor representations of multimodal data. It is noteworthy that the low-rank decomposition matrices can be used to recover the full-weight tensor originally used in tensor fusion (Zadeh *et al.*, 2017) and therefore dramatically reduce both the number of parameters and computation complexity involved in tensorization.

#### 2.2.3 Modality-specific representation learning

In addition to learning modality-invariant representations, CAMR further puts forward to learn modality-specific representations in private sub-space, which provide complementary information of the modality-invariant representations and can also be helpful in making survival prediction. Specifically, to learn discriminant modality-specific representations $h_{m}^{u}\in R^{d\times 1}$, we design a modality-specific representation learning network which contains three unique encoders to effectively exploit the specific information for each modality. The function of these encoders can be summarized as follows:

(8) hmu=Emufm;θmu,m∈P,G,C,

where $E_{m}^{u}\left(\cdot \right)\;$assigns respective parameters $\theta_{m}^{u}$ for each modality to capture unimodal characteristics.

In order to encourage the modality-invariant and -specific representations to learn distinctive characteristics of each modality, we introduce orthogonality constraints between them during the learning process of CAMR, as the common subspace and the private subspace are expected to be well separated. To be specific, a loss function is adopted to enforce orthogonality between the representations in the common and private subspaces of each modality, which can be calculated as:

(9) Lorth=∑m∈P,G,CHmc THmuF2,

where $H_{m}^{c}$ and $H_{m}^{u}$ denote the matrices whose rows represent the hidden vectors $h_{m}^{c}$ and $h_{m}^{u}$ for modality *m*, ${\left\|\cdot \right\|}_{F}^{2}$ is the squared Frobenius norm.

In addition to orthogonality constraints, to ensure that the modality-invariant and -specific representations work together to provide a comprehensive multimodal representation of the same patient, we also develop a reconstruction module to retain the modality information during representation learning. The reconstruction module is implemented by a decoder consisting of several FC layers, and is defined as follows:

(10) f^m=Dmhc⊕hmu,m∈P,G,C,

where$\;{\hat{f}}_{m}$ is the reconstruction of the input representation $f_{m}$, and $D_{m}$ denotes the reconstruction decoder. Accordingly, a reconstruction loss is adopted which encourages the cross-modality representation and modality-specific representations to recover the original representations of each modality:

(11) Lrec=∑m∈P,G,Cf^m-fm2,

where ${\left\|\cdot \right\|}_{2}$ is the squared $L_{2}$-norm.

#### 2.2.4 Survival prediction

To make survival prediction, CAMR first generates multimodal representation $h\in R^{4d\times 1}\;$by concatenating $h^{c}$ and $h_{m}^{u}$ thus taking full advantage of modality-invariant and modality-specific representations. This can be formulated as follows:

(12) h=hc⊕hPu⊕hGu⊕hCu.

After that, *h* is sent to a survival prediction module which contains four ReLU-activated FC layers including 900, 256, 64 and 15 nodes, respectively. Then, a Cox layer is used to perform Cox proportional hazards regression by following (Cheerla and Gevaert, 2019; Huang *et al.*, 2019). Accordingly, Cox partial likelihood loss with $l_{1}$ regularization is adopted to train survival prediction model, which can be described as follows:

(13) LCoxθ=-∑i:Ei=1h^θxi-log∑j:Tj>Tieh^θxi+λθ1,

where the values $E_{i}$, $T_{i}$ and $x_{i}$ denote the survival status, the survival time and the input data for each patient respectively, and ${\hat{h}}_{\theta }$ represents neural network model to predict the risk of survival, λ indicates a regularization hyperparameter to prevent the model from overfitting.

### 2.3 Training

The total loss of CAMR includes adversarial loss $L_{\mathrm{adv}}$, reconstruction loss $L_{\mathrm{rec}}$, orthogonality loss $L_{\mathrm{orth}}$ and the Cox partial likelihood loss $L_{\mathrm{Cox}}$. Accordingly, the final objective function of our proposed method is defined by a linear combination of the above loss terms as:

(14) Ltotal=LCox+αLadv+βLrec+ γLorth,

where, α, β and γ represent the weights of $L_{\mathrm{adv}}$, $L_{\mathrm{rec}}$ and $L_{\mathrm{orth}}$. Through the experiments, the value of α,  β and γ are set to 0.6, 0.8 and 0.05, respectively. We optimize CAMR with the loss $L_{\mathrm{total}}$ during the training phase. PyTorch, a high-level neural network framework in python, is used to implement CAMR under Linux with GPU NVIDIA GeForce RTX 2080 Ti.

## 3 Results

### 3.1 Effectiveness of CAMR architecture

#### 3.1.1 Ablation experiments on CAMR

To verify the effectiveness of CAMR, we analyze the performance of different losses during the training procedure. The results are presented in Table 1 and Supplementary Tables S2 and S3, where CAMR-B0 represents CAMR only using Cox partial likelihood loss, +L_*_ indicates the specific loss is added to CAMR-B0 and$\;L_{\mathrm{all}}$ means that we use all the losses in Equation (14). For fair comparison, we use the same survival prediction module as described in Section 2.2.4 in all the experiments.

**Table 1.** Ablation experiments of CAMR on LGG dataset

Methods	C-index	AUC
CAMR-B0(with only$\;L_{\mathrm{Cox}})$	0.795 ± 0.013	0.805 ± 0.023
CAMR-B1(+$L_{\mathrm{adv}})$	0.818 ± 0.018	0.833 ± 0.030
CAMR-B2(+$L_{\mathrm{rec}})$	0.808 ± 0.019	0.821 ± 0.027
CAMR-B3(+$L_{\mathrm{orth}})$	0.799 ± 0.016	0.815 ± 0.028
CAMR-B4(+$L_{\mathrm{adv}}$+$L_{\mathrm{orth}})$	0.822 ± 0.022	0.846 ± 0.013
CAMR-B5(+$L_{\mathrm{adv}}$+$L_{\mathrm{rec}})$	0.829 ± 0.027	0.853 ± 0.034
CAMR-B6(+$L_{\mathrm{rec}}$+$L_{\mathrm{orth}})$	0.818 ± 0.013	0.841 ± 0.018
CAMR($L_{\mathrm{all}})$	0.841 ± 0.020	0.889 ± 0.017

The following observations can be drawn from results on LGG in Table 1. We can find that CAMR-B0 obtains the worst performance among all the methods, suggesting that the existence of modality gaps can largely hamper multimodal information integration of histopathology images and genomic data. In contrast, we can find that aligning cross-modality representation in the common subspace (+$L_{\mathrm{adv}}$) plays a key role in addressing modality gap problem and thus improving the performance of survival prediction. For example, CAMR-B1 outperforms CAMR-B0 with 2.3% and 2.8% improvements on C-index and AUC, respectively. Compared with CAMR-B1, both CAMR-B4 and CAMR-B5 exhibit better performance, suggesting that the proposed orthogonality loss and reconstruction loss aid in effective feature learning of modality-invariant and -specific representations. Besides, CAMR-B2 and CAMR-B3 have better performance than CAMR-B0, which illustrates that orthogonal loss and reconstructed loss are also helpful for improving predictive performance when working alone. More importantly, we can infer from Table 1 that CAMR with all losses obviously improves the values of C-index and AUC, and achieves the best performance among all these methods. Besides, the results of BRCA and LUSC are provided in Supplementary Tables S2 and S3, which evidently illustrate the advantage of CAMR in making survival prediction of different cancer types. Also, it is of note that CAMR’s performance is robust to the choice of target modality and the detailed information is provided in Supplementary Methods and Supplementary Table S4. In summary, these results clearly demonstrate that CAMR is superior in efficiently learning more discriminative multimodal representations by successfully mitigating the detrimental gaps amongst different modalities.

To further verify the effectiveness of CAMR, Kaplan–Meier curves of all above methods on LGG are illustrated in Supplementary Figure S1. Specifically, by following previous work, we concatenate predicted risk values from the test sets in 5-fold cross-validation, which are then plotted against the respective survival time. Patients of each dataset are divided into low and high-risk groups based on the on the median of risk indices as the threshold (Ning *et al.*, 2021a). We can find that CAMR successfully divides the LGG patients into low and high-risk groups with optimal patient stratification (*P* = 2.2204e−16). In addition, we plot Kaplan–Meier curves of BRCA and LUSC in Supplementary Figures S2 and S3, which also corroborate the synergistic effect of multiple losses introduced in this study and the power of CAMR for improving the performance of cancer survival prediction.

#### 3.1.2 Evaluation of CMFM

To assess the capability of CMFM, we adopt four different configurations of experiments as follows: (i) Concat: concatenation using modality-invariant representations as input, serving as a baseline model for comparison; (ii) LMF: LMF using modality-invariant representations as input; (iii) CMFM: CMFM using modality-invariant representations as input; and (iv) CMFM*: further combination of the fused cross-modality representations generated by CMFM and the modality-specific representations.

From the experiment results reported in Table 2, we can clearly see that CMFM compares favorably against both Concat and LMF on LGG. For example, the AUC value obtained by CMFM is 0.871, with 4.8% and 2.3% improvement over Concat and LMF, respectively. In addition, we list the comparative results of different fusion methods on BRCA and LUSC in Supplementary Tables S5 and S6, in which CMFM also consistently outperforms the competing fusion approaches. Taken together, these results highlight that CMFM is capable of exploring complicated relations among modality-invariant representations and fusing them into more expressive and discriminative cross-modality representation. Besides, CMFM* obtains higher C-index and AUC values than CMFM, suggesting that the fused cross-modality representations generated by CMFM can be further strengthened by the complimentary information retrieved from modality-specific representation learning in CAMR.

**Table 2.** Evaluation of CMFM on LGG dataset

Methods	C-index	AUC
Concat	0.797 ± 0.011	0.823 ± 0.035
LMF	0.815 ± 0.015	0.848 ± 0.023
CMFM	0.828 ± 0.016	0.871 ± 0.016
CMFM*	0.841 ± 0.020	0.889 ± 0.017

### 3.2 Comparison with existing methods

We conduct a systematic performance evaluation by comparing CAMR with traditional methods En-Cox (Yang and Zou, 2013), LASSO-Cox (Tibshirani, 1997), RSF (Ishwaran *et al.*, 2008) GCCA (Subramanian *et al.*, 2021) and MLW-gcForeast (Dong *et al.*, 2019), as well as deep-learning methods MDNMMN (Sun *et al.*, 2019), DeepSurv (Katzman *et al.*, 2018), Pathomic Fusion (Chen *et al.*, 2022) and GPDBN (Wang *et al.*, 2021b). It is noteworthy that all above prediction methods adopt the same input features throughout the experiment to obtain fair comparison. As shown in Table 3, by integrating multimodal information, all these methods can yield fairly good results. Besides, we can find that GCCA and MLW-gcForest compare favorably with other traditional methods. For example, MLW-gcFores achieves C-index of 0.603 on LUSC and outperforms En-Cox by 1.4%. At the same time, deep learning-based methods in general have higher performance than traditional methods. For example, Pathomic Fusion obtains a better C-index value of 0.821 than all traditional methods on LGG. More importantly, compared with other deep learning-based methods, our proposed method shows enhanced performance and achieves C-index of 0.841 (LGG), 0.780 (BRCA) and 0.650 (LUSC), which outperforms the second best method by 2.0%, 4.3% and 2.0%, respectively. In addition to C-index value, we draw box plots of the AUC values of different methods in Supplementary Figs S4 and S6, in which CAMR also provides consistently better performance than other approaches. Taken together, the above results demonstrate that CAMR can successfully facilitate comprehensive integration of multimodal information and therefore remarkably improve the performance of cancer survival prediction.

**Table 3.** Performance comparison of CAMR and other methods using C-index value

Methods		C-index		
		LGG	BRCA	LUSC
Traditional	En-Cox	0.779 ± 0.016	0.702 ± 0.041	0.589 ± 0.017
	Lasso-Cox	0.785 ± 0.014	0.698 ± 0.055	0.567 ± 0.027
	RSF	0.790 ± 0.022	0.626 ± 0.040	0.542 ± 0.029
	GCCA	0.793 ± 0.026	0.708 ± 0.039	0.598 ± 0.021
	MLW-gcForest	0.798 ± 0.021	0.705 ± 0.036	0.603 ± 0.023
Deep learning	MDNMMN	0.804 ± 0.025	0.721 ± 0.039	0.614 ± 0.039
	DeepSurv	0.805 ± 0.017	0.715 ± 0.033	0.630 ± 0.060
	Pathomic fusion	0.821 ± 0.027	0.737 ± 0.039	0.620 ± 0.043
	GPDBN	0.818 ± 0.028	0.726 ± 0.032	0.618 ± 0.045
	CAMR	0.841 ± 0.020	0.780 ± 0.048	0.650 ± 0.037

**Fig. 2.**
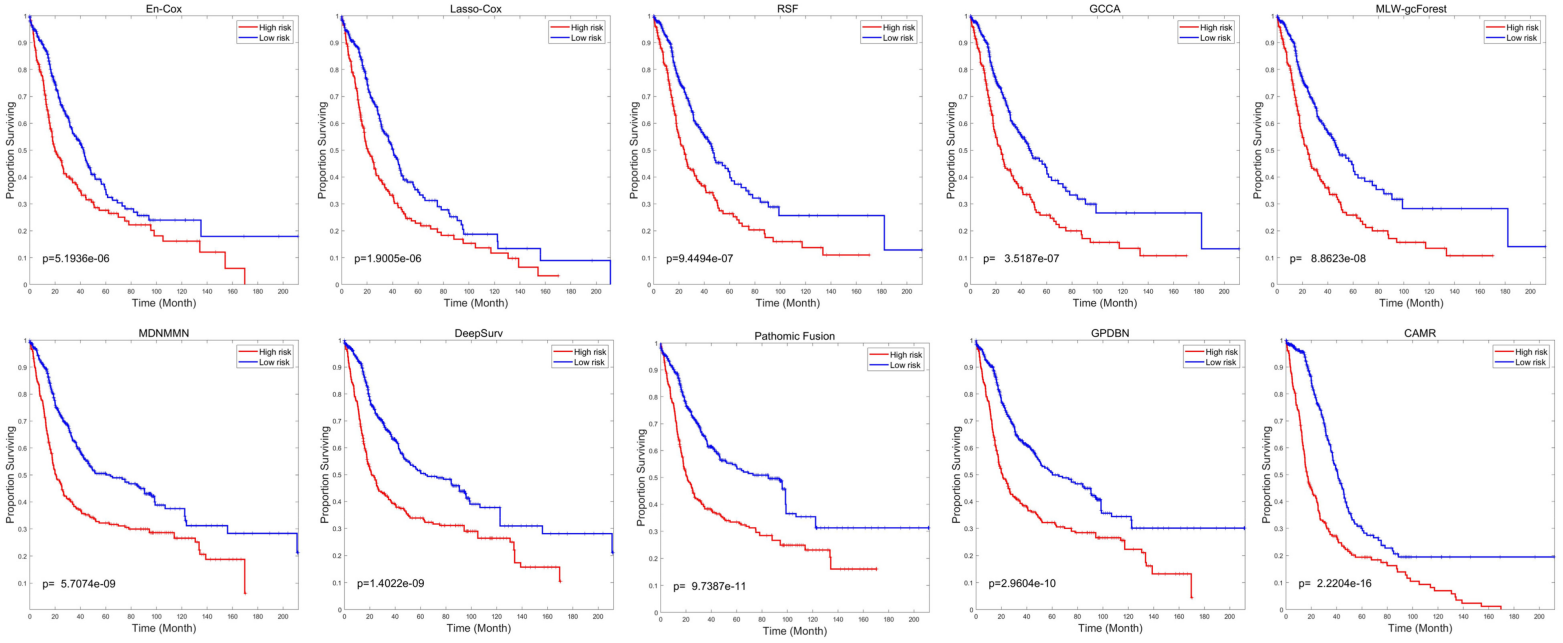
Performance comparison of CAMR and other methods on LGG dataset using Kaplan–Meier curve

Furthermore, Kaplan–Meier curves of all investigated methods on LGG are plotted in Figure 2. Among traditional methods, RSF can provide good prognostic prediction with a *P*-value of 9.4494e−9. Besides, compared to RSF, MLW-gcForest has better survival prediction with a *P*-value of 8.8623e−8 on LGG. Meanwhile, deep learning-based method Pathomic Fusion and DeepSurv have competitive performance with a *P*-value of 9.7387e−11 and 1.4022e−9, respectively. Among the deep learning-based methods, our proposed method achieves the most significant *P*-value of 2.2204e−16, which further validates the capability of CAMR to improve the performance by reducing modality gaps. In addition, Kaplan–Meier curves on BRCA and LUSC are plotted in Supplementary Figs S7 and S8, in which CAMR also obtains preferable *P-*values of 5.2403e−13 and 1.9913e−11, respectively. In all, these results clearly confirm the effectiveness of our proposed method in predicting cancer survival.

### 3.3 Cox proportional hazards analysis

To verify the independent prognostic power of risk predicted by CAMR, we also perform univariate and multivariate Cox proportional hazards analysis for CAMR risk and other LGG clinical variables adopted by Zhang *et al.* (2020), including gender (female versus male), age, grade (G2 versus G3) and IDH mutation status (mutant versus wild-type). By following, the predicted risks from the test sets in 5-fold cross-validation are concatenated. As shown in Supplementary Table S7, univariate Cox proportional hazards analysis shows that CAMR risk is significantly associated with survival (*P* = 7.21e−11). Besides, we can observe that CAMR risk is identified as an important prognostic factor when correcting for other clinic variables by multivariate Cox proportional hazards analysis. To sum up, our proposed method has excellent predictive capacity and CAMR risk is an independent prognostic factor for LGG (*P* = 1.61e−8, hazard ratio 3.699, 95% CI 2.34–5.82).

### 3.4 Visualizing representations

**Fig. 3.**
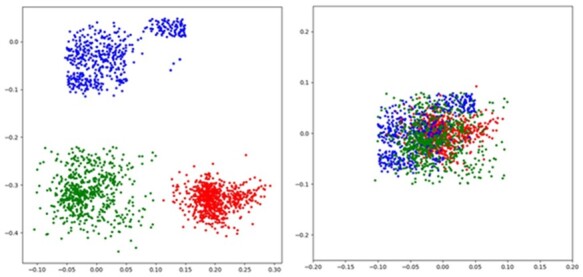
Visualization of t-SNE-mapped representations for LGG patients in the common subspace. The left and right images illustrate the representations of different modalities obtained without and with cross-modality representation alignment learning, respectively. The red dot represents histopathological image, the green dot represents gene expression and blue dot represents CNA (A color version of this figure appears in the online version of this article)

To visualize the learned representations in common subspace, we adopt t-SNE which is a popular algorithm to squeeze high dimensional features into a 2D space. In this way, the representations of different patients in the testing sets on LGG are shown in Figure 3. We can find from the left image that without cross-modality representation alignment learning, representations of different modalities are separated far from each other, indicating that they are not well mapped into a common subspace due to the existence of modality gaps. In contrast, the right image suggests that the issue of modality gaps is largely addressed by applying cross-modality representation alignment learning. This confirms that CAMR is able to obtain desired modality-invariant representations and therefore improve the performance of cancer survival prediction. Moreover, to validate the effectiveness of modality-specific representations, we visualize modality-invariant and -specific representations on LGG in Supplementary Figure S9. We can find from Supplementary Figure S9 that modality-invariant and -specific representations are in different subspaces, which means that these representations contain diverse information to provide more comprehensive representations. This result suggests that the predictive performance can be further enhanced by the complimentary information retrieved from modality-specific representation learning in CAMR.

## 4 Discussion

In this work, we present a novel deep learning-based method CAMR to make cancer survival prediction by effectively learning comprehensive multimodal representations. CAMR generates modality-invariant and -specific representations to provide a comprehensive view of the multimodal inputs. To reduce modality gaps, a cross-modality representation alignment learning network is introduced to generate modality-invariant representations in the common subspace. Then, CMFM is adopted to fuse modality-invariant representation by modeling complex relations across different modalities. Moreover, we leverage a modality-specific representation learning network to obtain modality-specific representations as complementary information for modality-invariant representations. The aforementioned experiment results illustrate that CAMR achieves excellent performance improvement over many existing methods by successfully narrowing modality gaps. Moreover, we analyze Kaplan–Meier curves and Cox proportional hazards to further verify the powerful capability of CAMR in multimodal survival prediction.

Recently, many approaches have been proposed to address the issue of cancer survival prediction, which can be divided into two categories: deep learning survival methods and non-deep learning ones. The differences between them can be summarized as follows. It is of note that deep learning-based methods have more powerful capability to effectively learn multimodal representations than non-deep learning methods, which plays a key role in helping to improve predictive performance. However, due to many parameters contained by deep learning-based methods, using deep learning-based methods to train survival prediction models sometimes may be expensive. Comparing with deep learning-based methods, non-deep learning methods have fewer parameters and computing resources required, hence the cost of model training reduces.

Although CNN has achieved great success in histopathological images, we choose pre-extract image features for further study. The reasons why we choose pre-extract features for a deep learning model can listed as follows. At first, limited by the computational resources, using CNN to extract features from histopathological images be expensive or even impractical (Ning *et al.*, 2021a). Secondly, the features retrieved by CNN may result in overfitting in small cohorts (Boehm *et al.*, 2022), which may limit the further improvement of performance of cancer survival prediction. At last, another advantage of pre-extract features is interpretability, which is helpful for further study.

Although CAMR has achieved good performance of cancer survival prediction, there is still large room for improvement. First, we will further improve CAMR by combining more modality data (e.g. miRNA expression and clinical data) that are also related to cancer survival. Secondly, we will try to explore other techniques such as cross-modal maximum mean discrepancy (Xu *et al.*, 2020) to align the distributions of cross-modality representations. Finally, to overcome the limited size of multimodal data adopted in this study, we plan to include more patients for improving cancer survival prediction in the future work. To sum up, by tackling the problem of modality gaps and integrating histopathology images and genomic data, we propose a novel cross-aligned representation learning method to learn efficient multimodal representations for predicting cancer survival, which can help to solve challenging prediction tasks and provide clues for further studies.

## Supplementary Material

btad025_Supplementary_Data
