# Supplementary material for: CAMR: cross-aligned multimodal representation learning for cancer survival prediction
Source: Bioinformatics. 2023 Jan 13;39(1):btad025. doi: 10.1093/bioinformatics/btad025 (PMC9857974; doi:10.1093/bioinformatics/btad025)
Supplement: btad025_Supplementary_Data [file btad025_supplementary_data.zip › btad025_Supplementary_Data/supplementary_final.pdf]

CAMR: Cross-Aligned Multimodal Representation Learning for cancer survival prediction

Supplementary Material

Xingqi Wu[1], Yi Shi[1], Minghui Wang[1] and Ao Li[1*]

1 Supplementary Methods

1.1 Evaluation metrics

In this work, we adopt Concordance Index (i.e. C-index) and AUC to assess the performance of CAMR. We use C-index to quantify the quality of the ranking at the patient level, which can be formulated as follows:

$$C - index = \frac{1}{N} \sum_{i \in \{1 \cdots N\}} \sum_{y_j > y_i} I\left(\hat{h}_\theta(x_i) > \hat{h}_\theta(x_j)\right) \quad (15)$$

where y_i denotes *i-th* patient's actual survival observation, *N* is the number of comparable pairs and $I(\cdot)$ represents the indicator function. Besides, the AUC is used to estimate the ranking quality at event-time (Shao *et al.*, 2020), and is defined as follows:

$$\text{AUC} = \frac{1}{num} \sum_{t \epsilon T} \sum_{y_i < t} \sum_{y_j > t} I\left(\hat{h}_\theta(x_i) > \hat{h}_\theta(x_j)\right) \quad (16)$$

where *num* denotes the cumulative number of comparable pairs calculated over all event times, *t* is the set of all possible event times in a dataset. The values of C-index and AUC range in an interval [0,1], with larger values corresponding to better predictive performance.

1.2 Choice of target modality

As described in Section 2.2.1, we choose gene expression as target modality for cross-modality distribution transformation. Technically speaking, it is also possible to adopt another modality, i.e., CNA or pathology images, as the target modality. To investigate whether the choice of target modality has an effect on survival prediction, we perform an experiment as followings:1) G,C→P: histopathological image is the target modality, gene expression and CNA are source modalities;2) G,P→C: CNA is the target modality, gene expression and histopathological image are source modalities;3) P,C→G: gene expression is the target modality, histopathological image and CNA are source modalities. As presented in Supplementary Table S3, we can find that using gene expression, histopathological image or CNA as target modality in general shows similar performance across three cancer types, suggesting CAMR's performance is robust to the choice of target modality. Besides using two discriminators to classify {G, P} and {G, C}, we utilize additional discriminator to classify {P, C}, and CAMR obtains C-index values of 0.832, 0.778 and 0.646 on LGG, BRCA and LUSC, respectively. This result suggests that predictive performance may not be further improved when using an additional discriminator to classify {P, C}. Therefore, it is good enough to choose one modality as the target modality and other modalities as source modalities, with the aim to transform the representation

distributions of the latter into those of the former.

1.3 Parameter analysis

We investigate the influence of the three hyperparameters alpha, beta, and gamma of CAMR on LGG. We fix a parameter and study the other two parameters. The results are listed in Supplementary Table S9-S11. From Table S9-S10, we can find that the performance of different values of alpha behaves closely, illustrating that CAMR is not sensitive to alpha when beta and gamma are fixed. Meanwhile, the performance shows the small-amplitude fluctuation, which shows its insensitivity to beta. Similarly, this trend is repeated on gamma.

References:

Shao,W. *et al.* (2020) Integrative Analysis of Pathological Images and Multi-Dimensional Genomic Data for Early-Stage Cancer Prognosis. *IEEE Trans. Med. Imaging*, **39**, 99–110.

1 Supplementary Tables

Table S1. Number of patients of each dataset

Dataset	LGG	BRCA	LUSC
samples	629	1015	491

Table S2. Ablation experiments of CAMR on BRCA dataset

Methods	C-index	AUC
CAMR-B0(with only L_{cox})	0.734±0.051	0.738±0.036
CAMR-B1(+L_{adv})	0.755±0.052	0.772±0.033
CAMR-B2(+L_{rec})	0.738±0.038	0.752±0.043
CAMR-B3(+L_{orth})	0.735±0.033	0.742±0.039
CAMR-B4(+L_{adv}+L_{orth})	0.759±0.048	0.774±0.054
CAMR-B5(+L_{adv}+L_{rec})	0.767±0.058	0.781±0.041
CAMR-B6(+L_{rec}+L_{orth})	0.740±0.042	0.758±0.058
CAMR(L_{all})	0.780±0.048	0.806±0.053

Table S3. Ablation experiments of CAMR on LUSC dataset

Methods	C-index	AUC
CAMR-B0(with only L_{cox})	0.611±0.032	0.641±0.036
CAMR-B1(+L_{adv})	0.628±0.034	0.663±0.017
CAMR-B2(+L_{rec})	0.621±0.028	0.651±0.033
CAMR-B3(+L_{orth})	0.616±0.033	0.742±0.039
CAMR-B4(+L_{adv}+L_{orth})	0.629±0.039	0.675±0.036
CAMR-B5(+L_{adv}+L_{rec})	0.642±0.060	0.689±0.041
CAMR-B6(+L_{rec}+L_{orth})	0.624±0.031	0.661±0.035
CAMR(L_{all})	0.650±0.037	0.701±0.033

Table S4. Performance comparison of different choices of target modality

Datasets	LGG		BRCA		LUSC	
Methods	C-index	AUC	C-index	AUC	C-index	AUC
G,C→P	0.835±0.011	0.878±0.020	0.771±0.063	0.795±0.025	0.642±0.041	0.696±0.037
G,P→C	0.833±0.021	0.877±0.029	0.767±0.062	0.792±0.048	0.639±0.038	0.692±0.026
P,C→G	0.841±0.020	0.889±0.017	0.780±0.048	0.806±0.053	0.650±0.037	0.701±0.033

Table S5. Evaluation of CMFM on BRCA dataset

Methods	C-index	AUC
Concat	0.724±0.056	0.733±0.036
LMF	0.750±0.055	0.769±0.043
MCFM	0.764±0.039	0.785±0.036
MCFM*	0.780±0.048	0.806±0.053

Table S6. Evaluation of CMFM on LUSC dataset

Methods	C-index	AUC
Concat	0.613±0.046	0.653±0.043
LMF	0.630±0.039	0.673±0.052
MCFM	0.641±0.044	0.692±0.038
MCFM*	0.650±0.037	0.701±0.033

Table S7. Hazard ratios for univariate and multivariate Cox proportional hazards analysis on LGG dataset

Variable	Univariate				Multivariate		
	Hazard ratio	95% CI	P value	C-index	Hazard ratio	95% CI	P value
CAMR	4.443	2.83-6.94	7.21e-11	0.841	3.699	2.34-5.82	1.61e-8
Gender	0.933	0.63-1.37	0.727	0.513	0.906	0.59-1.39	0.652
Age	1.928	1.22-3.04	0.005	0.644	1.53	0.96-2.45	0.074
Grade	2.941	1.83-4.70	7.00 e-6	0.664	2.41	1.54-4.02	1.97e-4
IDH mutation statues	1.703	1.10-2.62	0.016	0.521	1.378	0.73-2.58	0.317

Table S8. Performance comparison of different combinations of modality discriminators

Datasets	LGG		BRCA		LUSC	
Discriminators	C-index	AUC	C-index	AUC	C-index	AUC
{G,P}+{G,C}	0.841±0.020	0.889±0.017	0.780±0.048	0.806±0.053	0.650±0.037	0.701±0.033
{G,P}+{G,C}+{P,C}	0.832±0.018	0.881±0.025	0.778±0.051	0.798±0.042	0.646±0.035	0.697±0.029

{G,P}+{G,C} means that use discriminators to classify {G,P} and {G,C}, and {G,P}+{G,C}+{P,C} denotes that use discriminators to classify {G,P}, {G,C} and {P,C}.

Table S9. Parameters analysis when fixing gamma on LGG

alpha	0.4	0.4	0.4	0.6	0.6	0.6	0.8	0.8	0.8
beta	0.6	0.8	1.0	0.6	0.8	1.0	0.6	0.8	1.0
C-index	0.832±0.019	0.835±0.019	0.836 ±0.022	0.837±0.021	0.841±0.020	0.839±0.018	0.833±0.025	0.836±0.022	0.835±0.026
AUC	0.870±0.015	0.875±0.017	0.877±0.019	0.879±0.020	0.889±0.017	0.883±0.016	0.873±0.020	0.879±0.017	0.872±0.018

Table S10. Parameters analysis when fixing beta on LGG

alpha	0.4	0.4	0.4	0.6	0.6	0.6	0.8	0.8	0.8
gamma	0.0	0.05	0.1	0.0	0.05	0.1	0.0	0.05	0.1
C-index	0.831±0.019	0.834±0.015	0.833±0.026	0.829±0.027	0.841±0.020	0.835±0.018	0.832±0.025	0.835±0.023	0.833±0.016
AUC	0.864±0.013	0.873±0.018	0.870±0.017	0.853±0.034	0.889±0.017	0.876±0.016	0.870±0.020	0.876±0.017	0.873±0.018

Table S11. Parameters analysis when fixing alpha on LGG

beta	0.6	0.6	0.6	0.8	0.8	0.8	1.0	1.0	1.0
gamma	0.0	0.05	0.1	0.0	0.05	0.1	0.0	0.05	0.1
C-index	0.829±0.20	0.835±0.025	0.836 ±0.024	0.829±0.027	0.841±0.020	0.835±0.018	0.831±0.024	0.837±0.022	0.833±0.026
AUC	0.862±0.019	0.876±0.019	0.878±0.022	0.853±0.034	0.889±0.017	0.876±0.016	0.874±0.021	0.877±0.018	0.875±0.019

2 Supplementary Figures

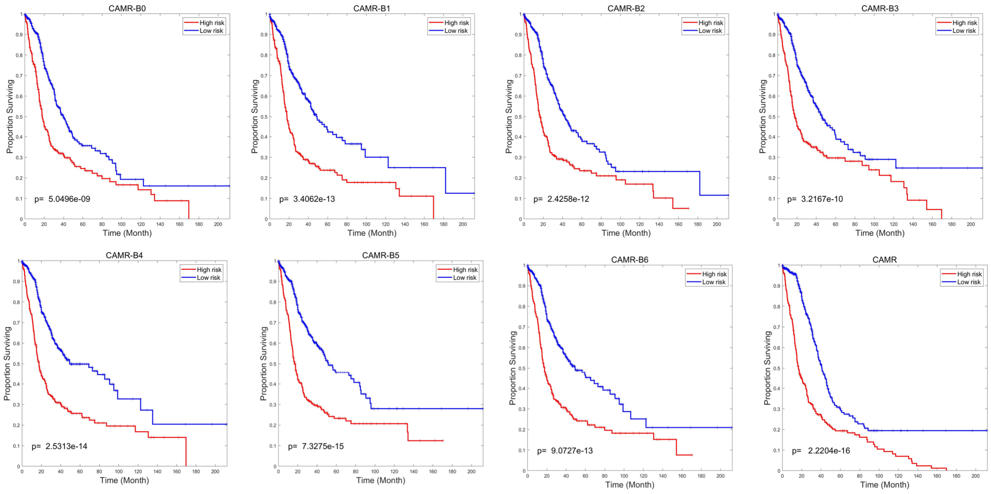

Fig. S1. Ablation experiments of CAMR on LGG dataset using Kaplan-Meier curve

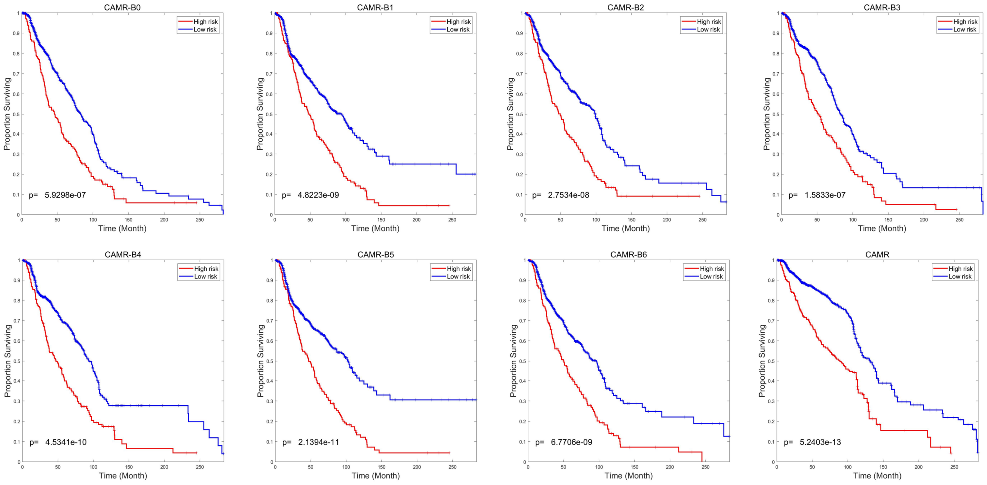

Fig. S2. Ablation experiments of CAMR on BRCA dataset using Kaplan-Meier curve

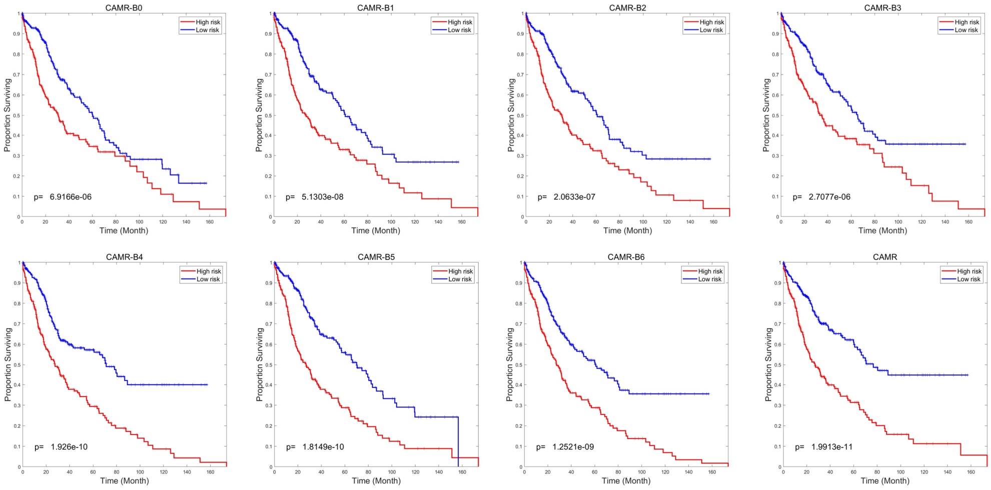

Fig. S3. Ablation experiments of CAMR on LUSC dataset using Kaplan-Meier curve

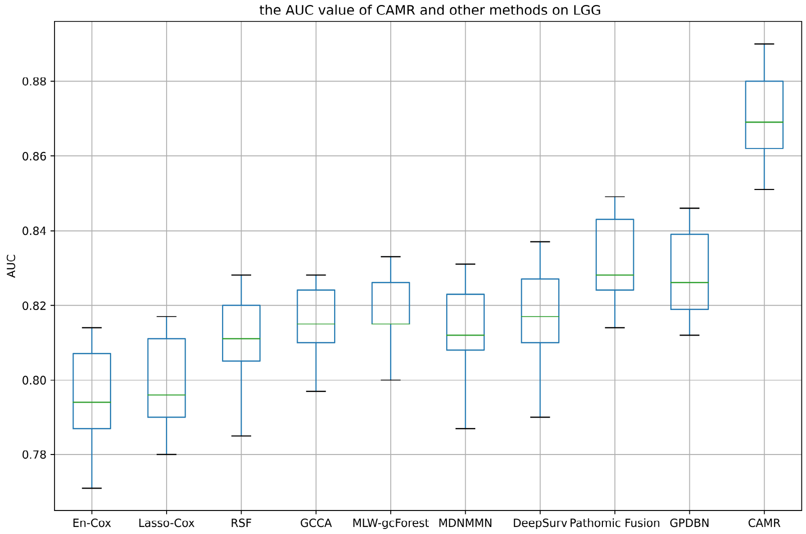

Fig. S4. Performance comparison of CAMR and other methods on LGG dataset using AUC value

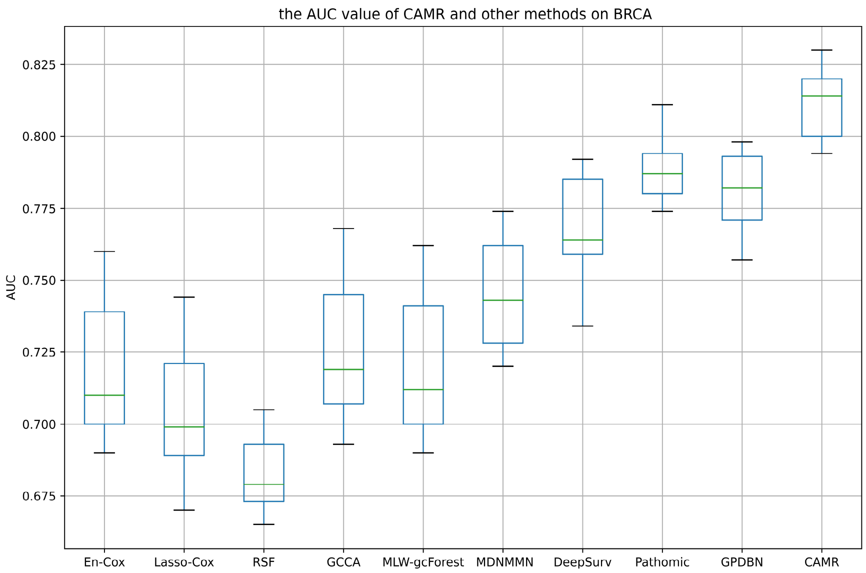

Fig. S5. Performance comparison of CAMR and other methods on BRCA dataset using AUC value

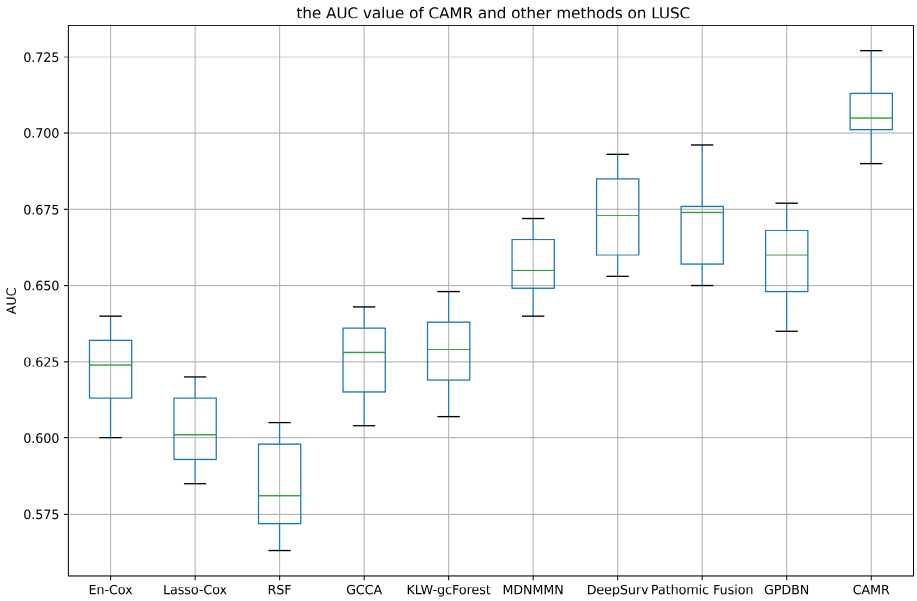

Fig. S6. Performance comparison of CAMR and other methods on LUSC dataset using AUC value

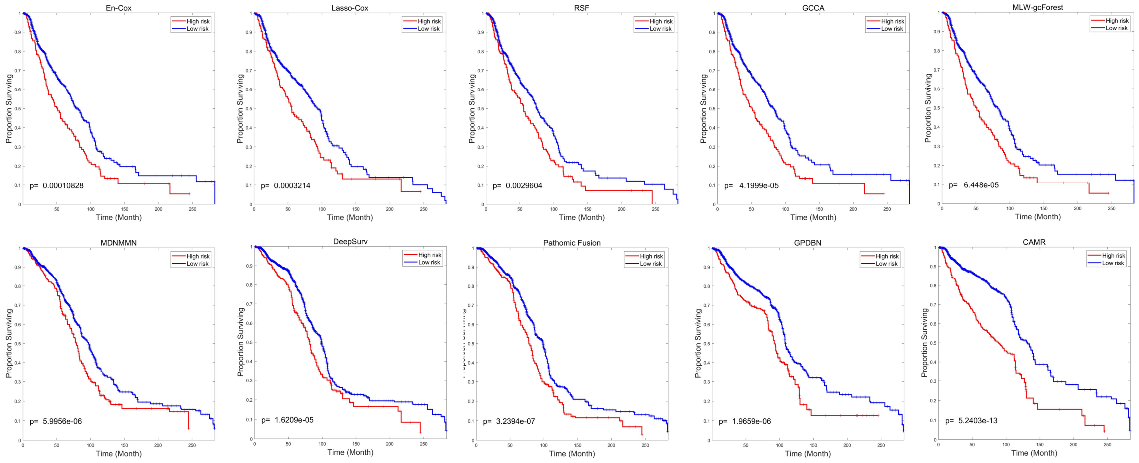

Fig. S7. Performance comparison of CAMR and other methods on BRCA dataset using Kaplan-Meier curve

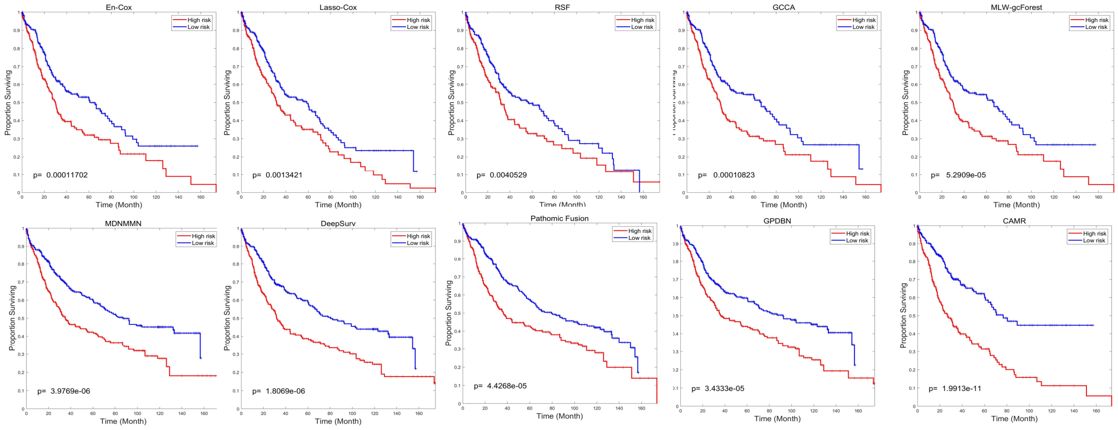

Fig. S8. Performance comparison of CAMR and other methods on LUSC dataset using Kaplan-Meier curve

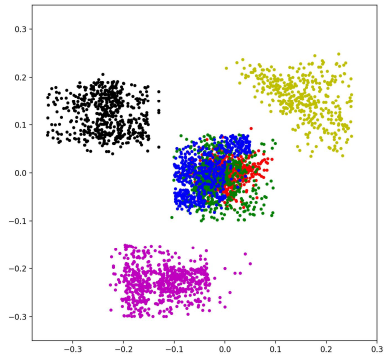

Fig. S9. Visualization of t-SNE- mapped modality-invariant and -specific representations for LGG patients. The red dot represents modality-invariant representations of histopathological image, the green dot represents modality-invariant representations of gene expression and blue dot represents modality-invariant representations of CNA. The purple dot represents modality-specific representations of histopathological image, the black dot represents s modality-specific representations of gene expression and yellow dot represents s modality-specific representations of CNA.
